# Enterovirus D68 Subclade B3 Strain Circulating and Causing an Outbreak in the United States in 2016

**DOI:** 10.1038/s41598-017-01349-4

**Published:** 2017-04-28

**Authors:** Guiqing Wang, Jian Zhuge, Weihua Huang, Sheila M. Nolan, Victoria L. Gilrane, Changhong Yin, Nevenka Dimitrova, John T. Fallon

**Affiliations:** 1 Department of Pathology, New York Medical College, Valhalla, New York, USA; 2 Department of Pathology and Clinical Laboratories, Westchester Medical Center, Valhalla, New York, USA; 3 Department of Pediatrics, Division of Infectious Disease, New York Medical College and Maria Fareri Children’s Hospital at Westchester Medical Center, Valhalla, New York, USA; 4 Philips Research North America, Cambridge, Massachusetts, USA

## Abstract

In 2014 the United States experienced a nationwide outbreak of Enterovirus D68 (EV-D68) infection. There were no confirmed cases of EV-D68 in 2015 and CDC was only aware of limited sporadic EV-D68 detection in the US in 2016. In this report, we analyzed 749 nasopharyngeal (NP) specimens collected in 2015 and 2016 from patients in the Lower Hudson Valley, New York using a previously validated EV-D68-specific rRT-PCR assay. EV-D68 was detected in none of 199 NP specimens collected in 2015, and in one of 108 (0.9%) samples from January to May and 159 of 442 (36.0%) samples from July to October 2016. Complete EV-D68 genome sequences from 22 patients in 2016 were obtained using a metagenomic next-generation sequencing assay. Comparative genome analysis confirmed that a new EV-D68 strain belonging to subclade B3, with 3.2–4.8% divergence in nucleotide from subclade B1 strains identified during the 2014 US outbreak, was circulating in the US in 2016 and caused an outbreak in the Lower Hudson Valley, New York with 160 laboratory-confirmed cases. Our data highlight the genetic variability and capacity in causing outbreak by diverse EV-D68 strains, and the necessity of awareness and more surveillance on their active circulation worldwide.

## Introduction

Enteroviruses in the family *Picornaviridae* are small, non-enveloped viruses with a single-stranded, positive-sense RNA genome of approximately 7.5 kilobases^1, 2^. The genus *Enterovirus* contains seven species, including enterovirus A to D and rhinovirus A to C that commonly cause human disease^2, 3^. Enterovirus D68 (EV-D68) was first recovered from patients with respiratory illness in California in 1962^4^ and was infrequently recognized until its recent emergence worldwide^5, 6^. In the United States, only 26 cases had been reported to the Centers for Disease Control and Prevention (CDC) from 1970 through 2005^7^.

The emergence of EV-D68 infection in patients with acute respiratory illness was first observed in the 2000’s in Asia, Europe and a few US states^5, 8–12^. In late summer/fall 2014, a nationwide outbreak of severe respiratory illness associated with EV-D68 was noticed from 49 US states with at least 1,153 confirmed cases^13, 14^. Also, 120 cases of acute flaccid myelitis (AFM), which coincided with this outbreak, were reported from 34 US states from August through December 2014^15–18^. An increase of EV-D68 cases was also documented in Canada, Eurasia and Australia in 2014^6, 19–23^. The US laboratories reported zero EV-D68 detections to CDC’s National Enterovirus Surveillance System (NESS) during the 2015 enterovirus season (summer and fall)^13^. CDC is only aware of limited sporadic EV-D68 detection in the US in 2016, although an increase of AFM cases from 21 in 2015 to 136 in 2016 was observed^18^. Most recently, an upsurge of EV-D68 infection in 2016 was noticed in several European countries, including the Netherlands^24^, France^25^ and Sweden^26^, with additional sporadic cases from the UK, Italy, Portugal and Germany^27^.

Genetic analysis of earlier EV-D68 strains collected between 1962 and 2011 has revealed that three lineages (lineages 1 to 3) or clades (clades A to C) of EV-D68 were circulating worldwide^5, 9, 28^. During and after the 2014 US outbreak, novel clade and subclades of EV-D68 strains, including subclades B1 to B3^15, 29–31^ and a new EV-D68 clade D^32^, have been identified and proposed. Sequence analyses of the EV-D68 strains collected in the US in 2014 showed two subclades (a major subclade B1 and a minor subclade B2) co-circulating at the time of the outbreak^14, 15, 29, 33^ (Wang *et al*., unpublished data). Midgley *et al*. reported that 92% of 606 cases analyzed had infection with subclade B1 strains, while subclade B2 strains accounted for 7% of EV-D68 cases in the 2014 outbreak^14^. One strain (US/KY/14-18953) is distantly related to subclades B1 and B2^14^ and has now been classified as clade D^32^. No EV-D68 strain belonging to clade A, clade C and subclade B3 has been identified in the 2014 US outbreak.

The Lower Hudson Valley, New York is located immediately north of New York City and consists of seven counties (Westchester, Putnam, Dutchess, Orange, Rockland, Ulster, and Sullivan counties) with approximately three million residents. During the 2014 nationwide EV-D68 outbreak, we reported 94 cases with severe respiratory illness from this region using an EV-D68-specific real-time reverse-transcription PCR (rRT-PCR)^34^ and a shotgun next-generation sequencing (NGS) assay^35^. We and other investigators also revealed that a novel clade of EV-D68 strain, now known as subclade B1, was responsible for the 2014 outbreak based on comparative genome analysis^15, 29, 33^. No EV-D68 case was confirmed in our facility in 2015. Recently, we noticed an increased number of patients with severe respiratory illness associated with EV-D68 infection in the Lower Hudson Valley, New York^36^. Our further investigation confirmed this regional outbreak of EV-D68 infection with 160 laboratory-confirmed cases in the US in 2016. In this report, we provided detailed epidemiology, viral genomics and clinical characteristics of EV-D68 cases identified from this outbreak. Strikingly, our comparative genome analysis suggests that a new EV-D68 strain belonging to subclade B3 was circulating in the US in 2016 and caused this outbreak.

## Results

### Detection of RhV/EV by FilmArray RP assay, 2014–2016

From January 2014 to October 2016, a total of 11,715 NP specimens were analyzed by FilmArray RP assay at the WMC Clinical Virology Laboratory. The overall positivity by the RP assay were 49.4%, with approximately 25% of these NP samples were positive to RhV/EV (Table 1). The overall RhV/EV positivity rates were comparable during the period from 2014 to 2016.

**Table 1:** Number of nasopharyngeal specimens examined by FilmArray RP during the period from 2014 to 2016.

Month & year	Total no. by RP	No. of positive^a^	Positivity (%)	No. of RhV/EV positive	RhV/EV positivity (%)
Jan-Dec 2014	3,762	1,769	47.0	917	24.4
Jan-Dec 2015	4,310	2,131	49.4	1,034	24.0
Jan-Oct 2016	3,643	1,882	51.7	985	27.0
Total	11,715	5,782	49.4	2,936	25.1

^a^One or more target(s) detected by the FilmArray RP assay.

**Figure 1:**
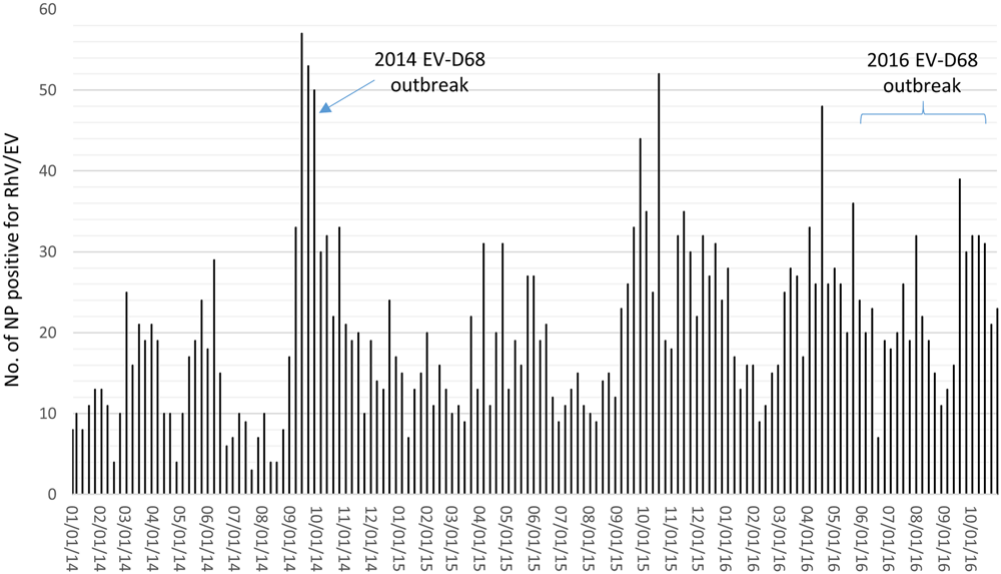
Weekly distribution of RhV/EV-positive nasopharyngeal (NP) specimens detected by the FilmArray Respiratory Panel assay, 2014 through October 2016.

The weekly distribution of RhV/EV-positive NP specimens from 2014 through October 2014 analyzed by the FilmArray RP assay is shown in Fig. 1. Typically, a minor peak of RhV/EV positives in the spring and early summer, followed by a major peak during autumn and early winter, were observed each year. The major peak observed in September and October 2014 corresponded to the US nationwide EV-D68 outbreak. A similar peak for RhV/EV positivity was also observed in 2015 from September through December. The spring-summer peak of RhV/EV positive in 2016 appeared higher than that in 2014, but the autumn-winter peak in 2016 was lower than that in 2014.

### Detection of EV-D68 by rRT-PCR, 2014–2016

In 2014, a total of 322 NP specimens were analyzed by rRT-PCR and DNA sequencing of partial VP1 gene and 5′ untranslated region; EV-D68 were detected in 95 patients as we previously reported^34, 35^. In a subsequent follow-up study, 199 NP specimens collected in September and October 2015 were examined retrospectively using the same rRT-PCR assay. One hundred eighty-six (n = 186) of these samples, including 140 RhV/EV-positive and 46 RhV/EV-negative NP specimens, were also examined by the NGS assay. None of these specimens in 2015 was positive to EV-D68 by either rRT-PCR or NGS assay, although multiple other respiratory viruses and bacteria were detected by the NGS assay (data not shown).

From January 1 to October 31, 2016, a total of 985 (27.0%) NP specimens from 861 patients were positive to RhV/EV by the RP assay. One hundred twenty-four (n = 124) NP specimens were duplicated or repeated and were removed from this analysis. Of 861 remaining RhV/EV-positive NP specimens, 416 (48.3%) were collected from January through May 2016 and 445 (51.7%) were collected from June through October 2016.

For EV-D68 rRT-PCR analysis, 108 of 416 (26.0%) representative RhV/EV-positive NP specimens collected from January through May 2016 were randomly selected, approximately 20 samples from each month. EV-D68 was detected by rRT-PCR in one of 108 (0.9%) RhV/EV-positive NP specimens collected from January through May 2016. The only EV-D68-positive sample was collected from a pediatric patient at the end of January 2016.

All but three (442 of 445, 99.3%) RhV/EV-positive NP specimens collected from June through October 2016 were selected and analyzed by rRT-PCR. One hundred fifty-nine (n = 159, 36.0%) of these NP specimens were positive to EV-D68 (Table 2).

**Table 2:** Number of nasopharyngeal specimens examined by the FilmArray Respiratory Panel and EV-D68 rRT-PCR from January to October 2016.

Month in 2016	No. of RhV/EV- positive	No. tested by rRT-PCR (%)	EV-D68 rRT-PCR		
			No. of negative	No. of positive	Positivity (%)
Jan-May	416	108 (26.0)	107	1	0.9
June	71	70 (98.6)	39	31	44.3
July	86	86 (100)	60	26	30.2
August	80	79 (98.8)	28	51	64.6
September	96	96 (100)	58	38	39.6
October	112	111 (99.1)	98	13	11.7
Total	861	550 (63.9)	390	160	29.1

**Figure 2:**
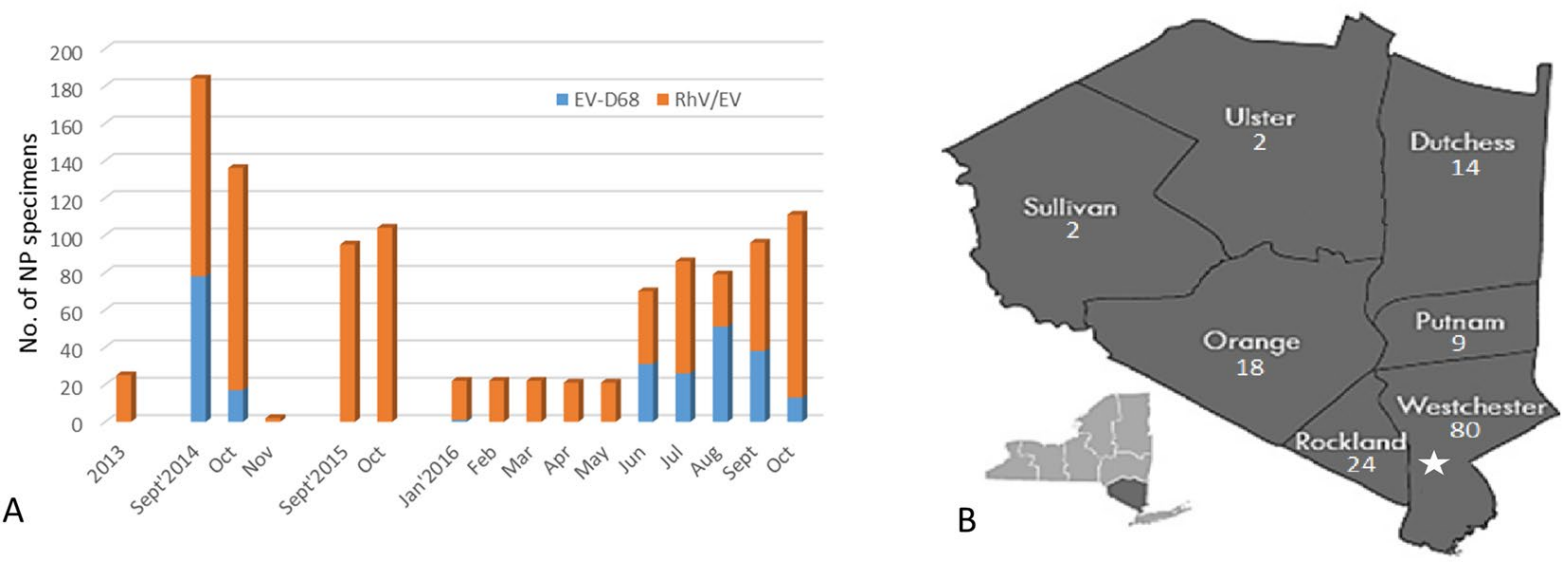
(**A**) Enterovirus D68 detected by an EV-D68-specific rRT-PCR assay in nasopharyngeal (NP) specimens collected from 2013 through October 2016; (**B**) Map of counties in the Lower Hudson Valley, New York and distribution on the number of patients with enterovirus D68 detected from respiratory samples in 2016. One hundred forty-nine (n = 149) of 160 (93.1%) confirmed cases in 2016 were from this region. The star indicates the site of the Westchester Medical Center. Map was adapted with permission from the New York State Department of Environmental Conservation website (http://www.dec.ny.gov/outdoor/7804.html).

Overall, EV-D68 was detected in respiratory samples of 254 patients by analyzing 1,096 NP specimens collected from 2013 to October 2016. Notably, EV-D68 was only detected in patients during the 2014 outbreak (n = 94) and the most recent upsurge in the Lower Hudson Valley in 2016 (n = 160). The temporal distribution of EV-D68 cases from 2013–2016 and geographic distribution of laboratory-confirmed EV-D68 cases in 2016 are shown in Fig. 2.

### Clinical characteristics of patients with EV-D68 in 2016

The median age of 160 patients from whom EV-D68 were detected was 3.2 years (3 weeks to 91 years) and 100 (62.5%) were male. Of these, 145 (90.6%) were pediatric patients with age ≤ 21 years and 15 (9.4%) were adult. The median age for pediatric patients was 2.6 years (3-week to 19-year old). Clinical data from 104 pediatric patients with specimens collected from June to mid-September were reviewed: the common clinical presentations included fever (n = 73, 70.2%), cough (n = 75, 72.1%), wheezing (n = 53, 51%), and increased work of breathing (n = 61, 58.6%). Thirty-seven (35.6%) had a prior history of asthma. Thirty-one of 104 (29.8%) pediatric patients required intensive care unit admission in 2016, comparable to 23 of 80 (28.8%) pediatric patients in 2014 (*p* = 0.8712). We had no AFM cases in 2014, but had two cases thus far in 2016; both were positive for EV-D68 in NP specimens and were in very young children (5 months and 21 months of age, respectively). Of 15 adult patients, eight had clinical data available and five of them had respiratory symptoms.

### Phylogenetic analysis of 2016 US EV-D68 strains

**Figure 3:**
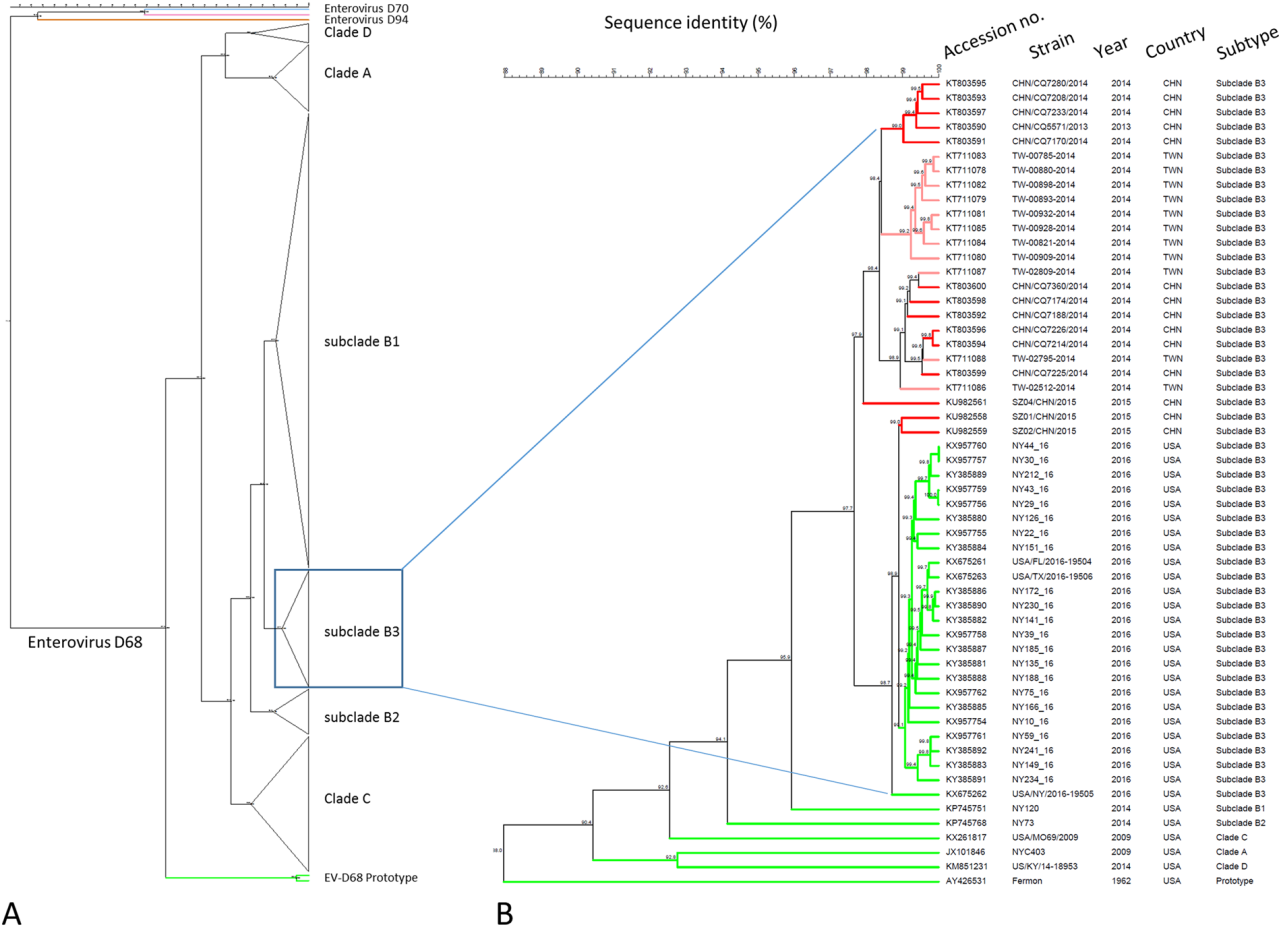
(**A**) Collapsed phylogenetic tree of enterovirus D68 based on nucleotide sequences of 341 complete or nearly complete genomes. Please refer to supplemental Figure S1 for a detailes phylogenetic tree with sequence accession numbers and strain information. Strains of EV-D70 and EV-D90 were used as outgroup; (**B**) An enlarged phylogenetic tree of EV-D68 subclade B3 strains (n = 50). Strains from China (CHN) and Taiwan (TWN) were shown in red and pink, respectively, whereas strains from the US were shown in green. One strain representing each of other subtypes (clades A, C and D, subclades B1 and B2, and prototype) was included for comparison. The numbers at each branch node are the % of nucleotide sequence identify.

A total of 22 complete or nearly complete EV-D68 genomes were obtained from respiratory specimens of patients during the 2016 outbreak in the Lower Hudson Valley, New York. Nine of these from our first NGS run were published recently^36^. Additional 13 were assembled from two subsequent NGS runs. Comparative genome analysis of 341 EV-D68 genomes available from the GenBank, including 22 from this study and three from 2016 patients in Florida, Texas and New York as reported by the CDC^37^, suggested that a novel EV-D68 strain belonging to subclade B3 was circulating in the US in 2016. As shown in Fig. 3 and Supplemental Figure S1, all 25 EV-D68 strains from the US in 2016 belonged to subclade B3, which were most closely related to two recent EV-D68 strains detected in Southern China in 2015 with 98.7–99.0% identity in nucleotides. The 23 additional EV-D68 strains from China and Taiwan all belonged to subclade B3 but were grouped into a separate subclutster.

The average genome divergence was 4.3% (ranging 3.2–4.8%) in nucleotides between EV-D68 subclade B3 strains from the US in 2016 and those of subclade B1 strains causing the 2014 US outbreak. This converted to an average of approximately 320 nucleotide difference per genome between subclades B3 and B1. The detailed nucleotide and amino acid sequence identity between subclade B3 and other subtypes of EV-D68 strains are summarized in Table 3.

**Table 3:** Nucleotide and amino acid sequence identity between subclade B3 and other subtypes of EV-D68 strains^a^.

EV-D68 clade	Sequence identity range (%)			
	Nucleotide, genome	Nucleotide, VP1	Amino acid, polyprotein	Amino acid, VP1
B3 vs. A	90.7–91.0	87.8–88.9	98.1–98.2	95.9–97.1
B3 vs. B1	95.5–95.8	95.4–96.5	99.0–99.3	98.6–99.4
B3 vs. B2	93.8–94.2	93.2–94.5	99.2–99.5	99.1–99.8
B3 vs. C	92.0–92.4	90.8–92.2	98.8–99.1	96.6–97.8
B3 vs. D	89.7–89.9	97.3–88.8	98.2–98.3	96.4–97.4
B3 vs. Fermon	87.7–88.1	85.3–86.7	97.6–97.7	94.0–95.0

^a^Based on comparisons of 50 complete genomes of subclade B3 strains and representative strains from other clades and subclades of enterovirus D68. See Fig. 3 for a list of strains included.

**Figure 4:**
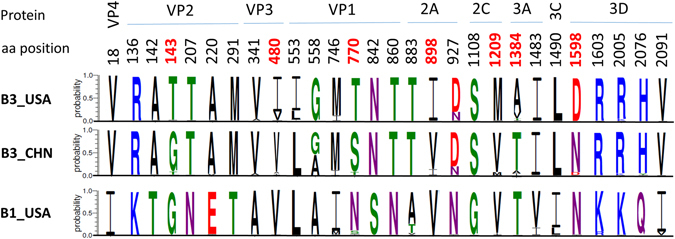
Amino acid (aa) polymorphisms of Enterovirus D68 subclade B3 based on the entire polypeptide sequences of approximate 2,190 aa. Twenty-eight amino acid polymorphisms were identified in subclade B3 strains from the US in 2016, as compared to those of subclade B1 strains from US in 2014. Of these, 7 amino acid substitutions that were first described in this report are highlighted in red with corresponding amino acid position numbers. See Fig. 3 for a list of subclade B3 strains included (n = 50). Twenty subclade B1 strains from New York in 2014 were used to generate the graphic presentation for the B1_USA. Amino acid position was based on the EV-D68 subclade B1 strain NY120 (accession #:KP745751).

A total of 28 amino acid polymorphisms were identified between subclade B3 strains from 2016 and B1 from 2014 in the US on basis of the complete polypeptide sequences of approximately 2,190 amino acids (Fig. 4). Among these, seven amino acid substitutions (T143, I480, T770, I898, M1209, A1384 and D1598) were first recognized in this study and were predominantly seen in subclade B3 strains from the US in 2016.

## Discussion

In 2014, the US experienced a nationwide outbreak of EV-D68 associated with severe respiratory illness. In addition to the 1,159 confirmed cases of EV-D68 infection reported from 49 states, CDC estimates that there were likely millions of mild EV-D68 infections during this outbreak for which people did not seek medical treatment and/or get tested. Surprisingly, no confirmed cases of EV-D68 infection was reported in 2015 and CDC has noticed only limited sporadic cases in the US as of October, 2016^13^. In this study, we reported an EV-D68 outbreak in the Lower Hudson Valley, New York in 2016. To the best of our knowledge, this represents the first report of an outbreak of EV-D68 infection in the US in 2016 with at least 160 laboratory-confirmed cases. An increase of AFM cases was co-incident with the EV-D68 nationwide outbreak in 2014, and in fact, the number of AFM cases reported to CDC in 2016 increased from 25 cases in 2015 to 108 cases as of October 2016. We speculate that the increased reports of AFM cases in 2016 may indicate many undetected or unreported EV-D68 cases in the US since EV-D68 infection is not a national notifiable disease, not routinely tested in clinical laboratories, and none of the FDA-cleared multiple PCR assays for respiratory pathogens differentiates EV-D68 from other rhinovirus and/or enteroviruses. Since sporadic EV-D68 cases in other US states have been confirmed by CDC, clinicians and public health agencies should be aware of the active circulation of EV-D68 in the US in 2016.

The 2016 EV-D68 outbreak in the Lower Hudson Valley, New York is most likely the first upsurge of EV-D68 infection in this region since 2014. This is evident by none of 199 NP specimens collected during the typical endemic months (September to October) in 2015 being positive for EV-D68, and EV-D68 was detected in only one of 108 (0.9%) randomly selected RhV/EV-positive NP specimens collected from January through May 2016. In contrast, 159 of 442 (36.0%) RhV/EV-positive NP specimens collected from June 1 through October 30, 2016 were positive to EV-D68 as confirmed by EV-D68-specific rRT-PCR and/or NGS assay. It is noteworthy that higher EV-D68 positivity rate was observed in 2016 among RhV/EV-positive NP specimens (36%, 159/422), compared to that seen during the 2014 outbreak (29.2%, 94/322). Nonetheless, we and other medical facilities in this region did not report significant increase in patients of emergency department visit or pediatric intensive care unit admission for respiratory disease during this regional EV-D68 outbreak. This can be in part explained by two factors as demonstrated in Fig. 1: the smaller number of RhV/EV-positive patients observed during typical peak RhV/EV months and the extended outbreak period from June through October in 2016.

Comparative analysis of 22 complete or nearly complete genomes of EV-D68 strains obtained in this study with those available from the GenBank confirms that the EV-D68 strains causing the 2016 outbreak in the Lower Hudson Valley, New York belong to a novel subclade B3^31^. Most recently, CDC reported three genome sequences from three patients, two with confirmed AFM from Florida and Texas and one with suspected AFM from New York, in 2016^37^. Our EV-D68 strains showed high identity (99.1–99.8% in nucleotide) to the three CDC strains; all 25 EV-D68 strains reported from the US in 2016 clustered into a tight sub-branch of the subclade B3 in the phylogenetic tree, suggesting that the same EV-D68 strain is circulating in the US in 2016, with 4.3% (ranging 3.2–4.8%) nucleotide divergence from subclade B1 strains causing the 2014 outbreak. An upsurge of subclade B3-associated EV-D68 infection in 2016 has recently been reported in the Netherlands^24^ and Sweden^26^. Due to the lack of complete genome sequences from the Netherlands and Sweden B3 strains, it is difficult to estimate the overall genetic similarity between B3 strains from the US and Europe. However, the capability of EV-D68 subclade B3 strains in causing outbreak in multiple countries in 2016 raises a concern on its future spread and potential to cause a global pandemic.

It is unknown if EV-D68 subclade B3 represents a strain totally different from the subclade B1 strain, or it constitutes a strain evolved from the subclade B1 strain causing the 2014 outbreak. Taraz *et al*. estimated that the mean rate of nucleotide substitution for the VP1 gene in EV-D68 was 6.2 × 10^−3^ substitutions per site/year^5^. Since the VP1 is the gene with highest genetic diversity in the EV-D68 genome, one may expect that the maximum nucleotide substitution would be no more than ~47 nucleotides per year over the approximately 7.5-kb genome. The average nucleotide divergence between B1 and B3 strains is 4.3%, which converts to difference of ~320 nucleotides. To reach this, it would take about 6.7 years to accumulate based on the substitution rate estimated above. Therefore, the subclade B3 strains circulating in the US and Europe in 2016 most likely represented a newly emerging virus strain, rather than a continuum of mutation from the subclade B1 strains responsible for the 2014 US outbreak. Limited evidence suggests that EV-D68 subclade B3 has emerged only in recent years, with the earliest complete genome retrieved in a 2013 sample from China (accession number: KT803590)^22^. No EV-D68 subclade B3 strains had been identified in the US until 2016, although it appeared to be predominant in patients of China and Taiwan in 2014^22, 31^.

Several clinical characteristics have been noticed among our patients with EV-D68 subclade B3 infection in 2016. First, subclade B3 was capable of causing severe respiratory illness as subclade B1 was during the 2014 outbreak. This is evident by the number of pediatric patients who required intensive care unit admission (31 of 104, 29.8% in 2016 versus 23 of 80, 28.8% in 2014, *p* = 0.8712). Second, the median age of pediatric patients was 2.8 years in 2016, versus 5 years in 2014, which might indicate partial immunity acquired through asymptomatic infection during the 2014 outbreak in older children. Third, subclade B3 was detected in 15 adult patients in 2016, while the majority remained to be pediatric patients. In contrast, all our cases in 2014 outbreak were pediatric patients. Last, we had no cases of AFM in 2014, but had two AFM cases with EV-D68 infection in 2016. Although the cause of these AFM cases has not yet determined, it is thought that many cases of EV-D68 induced disease including AFM in the US during the 2014 outbreak were associated with the newly emerging virus clades, including the subclade B1^15^. The appearance of these new variants may be a result of evolution of EV-D68 genome. It remains to be elucidated if the variable clinical presentations observed in this study were associated with an altered pathogenicity of the new subclade B3 virus or host factors, such as population structure and immunity.

A limitation of this study is that all the EV-D68 cases identified were based on NP specimens examined in a single institution, which might not reflect the true incidence of EV-D68 infection for the 2016 outbreak in this region. Also, only randomly selected NP specimens in September and October of 2015 (n = 199) and January through May of 2016 (n = 108) were analyzed for EV-D68. Given the high positivity rate (average of 29% and 36% in 2014 and 2016, respectively) of EV-D68 detected during the outbreak, the results from randomly selected specimens most likely represent the prevalence of EV-D68 among the population in the specific study period.

In conclusion, we report here a new EV-D68 subclade B3 circulating in the US that caused an outbreak in the Lower Hudson Valley, New York with 160 laboratory-confirmed cases in 2016. It is unclear to date if our observation indicates local or nationwide activity of EV-D68 in 2016. The lack of detected EV-D68 disease and relatively few AFM cases in 2015 compared with the recent increase of EV-D68-related respiratory illness in the Netherlands, Sweden and now the Hudson Valley, NY and increased AFM case reports in the US in 2016 potentially provides more evidence for a causative association between EV-D68 and AFM. This demonstrates the importance of providing accurate laboratory diagnostic testing for the detection of EV-D68 in clinical specimens. Clinicians, clinical laboratorians and public health agencies should be aware of the active circulation of EV-D68 and its clinical implications.

## Methods

### Patients

Patients included in this study are those with respiratory illness and/or other medical conditions who visited or hospitalized in multiple medical facilities in the Lower Hudson Valley in September and October 2015, and from January to October 2016. The majority of patients were those who visited the emergency department or hospitalized at the Maria Fareri Children’s Hospital at Westchester Medical Center (WMC), a tertiary teaching hospital 25 miles north of New York City serving patients mainly in the Lower Hudson Valley, New York. All patients included had a nasopharyngeal swab or aspirate (NP) specimen collected and tested for multiple respiratory pathogens at the WMC Clinical Virology Laboratory.

For study subjects, only leftover nasopharyngeal swab specimens collected for testing of respiratory pathogens at the WMC Laboratory were used in this study. Methods were carried out in accordance with the Department of Health and Human Services CFR 45 Part 46 Protection of Human Subjects. The New York Medical College Institutional Review Board approved all experimental protocols of this study and granted a waiver for informed consent from patients.

### Level of biocontainment

Enterovirus D68 in the *Picornaviridae* family belongs to biological risk group 2 microorganism per the US National Institute of Health (http://osp.od.nih.gov/office-biotechnology-activities/biosafety/biosafety-guidance). This study was carried out in certified biosafety level 2 laboratories in accordance with current CDC and institutional biosafety guidelines. All primary clinical specimens were processed in a Class II biosafety cabinet. No viral culture was performed.

### FilmArray Respiratory panel assay

NP specimens were collected into tubes each with 1-mL of viral transport medium (Diagnostic Hybrid, San Diego, CA) and were analyzed for the presence of rhinovirus/enterovirus (RhV/EV) and other respiratory pathogens using the FilmArray Respiratory Panel (RP) kit (version 2.0,) on the FilmArray instrument (v2.0, BioFire Inc., Salt Lake City, UT).

### Selection of specimen for EV-D68 analysis

NP specimens were selected by the following criteria and further analyzed using an EV-D68-specific rRT-PCR and NGS: (1) all RhV/EV-positive NP specimens by the RP assay that were collected between June 1, 2016 and October 31, 2016; (2) randomly selected representative RhV/EV-positive NP specimens that were collected from January through May 2016 (approximately 25%); (3) randomly selected representative RhV/EV-negative NP specimens that were collected from January to October 2016. For comparison, all RhV/EV-positive NP specimens collected in September and October 2015 were also included. For patients with multiple NP specimens examined during the study period, only the first NP specimen was included in the final analysis for EV-D68.

### EV-D68 real-time RT-PCR assay

The EV-D68 rRT-PCR was performed as described previously^34^. Total RNA was extracted from leftover NP specimens (≤150 µl) using the EZ1 Virus Mini kit, v2.0 (Qiagen, Valencia, CA) on the EZ1 Advanced XL instrument (Qiagen) without carrier RNA. RNA was eluted in 60 µl of buffer. A single-step reverse transcription PCR was carried out on either ABI 7500 Fast Dx or ViiA7 real-time PCR system (Life Technologies, Carlsbad, CA). A positive EV-D68 was defined for a sample exhibiting an exponential amplification and with a cycle threshold (Ct) of ≤40.0.

### Metagenomic Next-Generation Sequencing

Representative RhV/EV-positive and negative NP specimens were analyzed by a shotgun metagenomic NGS using the MiSeq system (Illumina, San Diego, CA) as described previously^29, 35^, with an exception that paired-end sequencing was performed as 2 × 76 base pairs (bp). Raw sequence reads were aligned and curated using a reference genome (strain NY120, accession number KP745751) from a 2014 patient.

### Phylogenetic analysis

Complete or nearly complete genomes from 341 EV-D68 strains, including 22 obtained from this outbreak and three from patients in Florida, Texas and New York as reported by the CDC in 2016^37^, were included in comparative genome analysis. Sequences downloaded from the GenBank or obtained in this study were aligned, and a phylogenetic tree based on genome sequences was constructed using the unweighted pair-group method with arithmetic averages (UPGMA) clustering method with the BioNumerics software (version 7.6, Applied Maths, Belgium). The amino acid polymorphisms and substitutions identified by sequence analysis were plotted on graphs using the WebLogo 3.5.0^38^.

### Statistical analysis

Fisher’s exact test was used to compare the pediatric ICU admission rate between 2014 and 2016 using the Prism GraphPad software (version 7, La Jolla, CA).

### Nucleotide sequence accession numbers

Complete genomes of 22 EV-D68 strains from this 2016 outbreak have been deposited to the NCBI GenBank database with accession numbers KX957754 to KX957762^36^, and KY385880 to KY385892.

## Electronic supplementary material

Supplemental Figure 1

## References

[1] Muir P (1998). Molecular typing of enteroviruses: current status and future requirements. Clin Microbiol Rev.

[2] King, A. M. Q. B. F., Christian, P. *et al*. In *Virus Taxonomy: Classification and Nomenclature of Viruses: Ninth Report of the International Committee on Taxonomy of Viruses* (eds A. M. Q. Adams King, M. J. Carstens, E. B. & E. J. Lefkowitz) 855–880 (Academic Press 2012).

[3] International Committee on Taxonomy of Viruses (ICTV). *Virus taxonomy*, *2015 release*, http://www.ictvonline.org/virustaxonomy.asp (2015).

[4] Schieble JH, Fox VL, Lennette EH (1967). A probable new human picornavirus associated with respiratory diseases. Am J Epidemiol.

[5] Tokarz R (2012). Worldwide emergence of multiple clades of enterovirus 68. J Gen Virol.

[6] Holm-Hansen CC, Midgley SE, Fischer TK (2016). Global emergence of enterovirus D68: a systematic review. Lancet Infect Dis.

[7] Khetsuriani N (2006). Enterovirus surveillance–United States, 1970-2005. MMWR Surveill Summ.

[8] Imamura T (2011). Enterovirus 68 among children with severe acute respiratory infection, the Philippines. Emerg Infect Dis.

[9] Meijer A (2012). Emergence and epidemic occurrence of enterovirus 68 respiratory infections in The Netherlands in 2010. Virology.

[10] CDC (2011). Clusters of acute respiratory illness associated with human enterovirus 68–Asia, Europe, and United States, 2008–2010. MMWR Morb Mortal Wkly Rep.

[11] Imamura T, Oshitani H (2014). Global reemergence of enterovirus D68 as an important pathogen for acute respiratory infections. Rev Med Virol.

[12] Imamura T (2013). Molecular evolution of enterovirus 68 detected in the Philippines. PLoS One.

[13] CDC. *Non-Polio Enterovirus: Enterovirus D68*, http://www.cdc.gov/non-polio-enterovirus/about/ev-d68.html (Accessed on October 5, 2016) (2016).

[14] Midgley CM (2015). Severe respiratory illness associated with a nationwide outbreak of enterovirus D68 in the USA (2014, 3, 879–887): a descriptive epidemiological investigation. Severe respiratory illness associated with a nationwide outbreak of enterovirus D68 in the USA (2014): a descriptive epidemiological investigation. Lancet Respir Med.

[15] Greninger AL (2015). A novel outbreak enterovirus D68 strain associated with acute flaccid myelitis cases in the USA (2012-14): a retrospective cohort study. Lancet Infect Dis.

[16] Messacar K (2015). A cluster of acute flaccid paralysis and cranial nerve dysfunction temporally associated with an outbreak of enterovirus D68 in children in Colorado, USA. Lancet.

[17] Sejvar JJ (2016). Acute Flaccid Myelitis in the United States, August-December 2014: Results of Nationwide Surveillance. Clin Infect Dis.

[18] CDC. Acute Flaccid Myelitis, https://www.cdc.gov/acute-flaccid-myelitis/ (Last updated: March 1, 2017; accessed on March 15, 2017).

[19] Skowronski, D. M. *et al*. Systematic community- and hospital-based surveillance for enterovirus-D68 in three Canadian provinces, August to December 2014. *Euro Surveill* **20**, doi:10.2807/1560-7917.ES.2015.20.43.30047 (2015). 10.2807/1560-7917.ES.2015.20.43.30047 26804195

[20] Poelman R (2015). European surveillance for enterovirus D68 during the emerging North-American outbreak in 2014. J Clin Virol.

[21] Levy A (2015). Enterovirus D68 disease and molecular epidemiology in Australia. J Clin Virol.

[22] Xiao Q (2015). Prevalence and molecular characterizations of enterovirus D68 among children with acute respiratory infection in China between 2012 and 2014. Sci Rep.

[23] Zhang T (2015). Enterovirus D68-associated severe pneumonia, China, 2014. Emerg Infect Dis.

[24] Knoester M (2017). Upsurge of Enterovirus D68, the Netherlands, 2016. Emerg Infect Dis.

[25] Antona, D. *et al*. Severe paediatric conditions linked with EV-A71 and EV-D68, France, May to October 2016. *Euro Surveill* **21**, doi:10.2807/1560-7917.es.2016.21.46.30402 (2016). 10.2807/1560-7917.ES.2016.21.46.30402 PMC5144948 27918268

[26] Dyrdak, R. *et al*. Outbreak of enterovirus D68 of the new B3 lineage in Stockholm, Sweden, August to September 2016. *Euro Surveill* **21**, doi:10.2807/1560-7917.es.2016.21.46.30403 (2016). 10.2807/1560-7917.ES.2016.21.46.30403 PMC5144949 27918255

[27] European Centre for Disease Prevention and Contorl. Rapid risk assessment - Enterovirus detections associated with severe neurological symptoms in children and adults in European countries. Auguest 8, 2016. Stockholm:ECDC, 1–9 (2016).

[28] Ikeda T (2012). Acute respiratory infections due to enterovirus 68 in Yamagata, Japan between 2005 and 2010. Microbiol Immunol.

[29] Huang W (2015). Whole-genome sequence analysis reveals the Enterovirus D68 isolates during the United States 2014 outbreak mainly belong to a novel clade. Sci Rep.

[30] Lau SK (2016). Enterovirus D68 infections associated with severe respiratory illness in elderly patients and emergence of a novel clade in Hong Kong. Sci Rep.

[31] Gong YN (2016). Molecular evolution and the global reemergence of enterovirus D68 by genome-wide analysis. Medicine (Baltimore).

[32] Du J (2015). Analysis of Enterovirus 68 Strains from the 2014 North American outbreak reveals a new clade, indicating viral evolution. PLoS One.

[33] Brown BA, Nix WA, Sheth M, Frace M, Oberste MS (2014). Seven strains of Enterovirus D68 detected in the United States during the 2014 severe respiratory disease outbreak. Genome Announc.

[34] Zhuge J (2015). Evaluation of a Real-Time Reverse Transcription-PCR Assay for Detection of Enterovirus D68 in Clinical Samples from an Outbreak in New York State in 2014. J Clin Microbiol.

[35] Huang W (2016). Assessing next-generation sequencing and 4 bioinformatics tools for detection of Enterovirus D68 and other respiratory viruses in clinical samples. Diagn Microbiol Infect Dis.

[36] Huang W (2016). Complete genome sequences of nine Enterovirus D68 strains from patients of the Lower Hudson Valley, New York, 2016. Genome Announc.

[37] Ng TF (2016). Detection and Genomic Characterization of Enterovirus D68 in Respiratory Samples Isolated in the United States in 2016. Genome Announc.

[38] Crooks GE, Hon G, Chandonia JM, Brenner SE (2004). WebLogo: a sequence logo generator. Genome Res.

