# Supplementary material for: Enterovirus D68 Subclade B3 Strain Circulating and Causing an Outbreak in the United States in 2016
Source: Sci Rep. 2017 Apr 28;7:1242. doi: 10.1038/s41598-017-01349-4 (PMC5430842; doi:10.1038/s41598-017-01349-4)
Supplement: Supplementary file 1 — Supplemental Figure 1 [file 41598_2017_1349_MOESM1_ESM.pdf]

ENTEROVIRUS D68 SUBCLADE B3 STRAIN CIRCULATING AND CAUSING AN OUTBREAK IN THE UNITED STATES IN 2016

Guiqing Wang[1, 2, *], Jian Zhuge[2], Weihua Huang[1], Sheila M. Nolan[3], Victoria L. Gilrane[1], Changhong Yin[1], Nevenka Dimitrova[4], John T. Fallon[1, 2]

[1] Department of Pathology, New York Medical College, Valhalla, New York, USA;

[2] Department of Pathology and Clinical Laboratories, [3] Department of Pediatrics, Division of Infectious Disease, New York Medical College and Maria Fareri Children's Hospital at Westchester Medical Center, Valhalla, New York, and [4] Philips Research North America, Cambridge, Massachusetts, USA.

SUPPLEMENTAL MATERIALS

Supplemental Figure S1. Complete phylogenetic tree based on complete or nearly complete genomes of 341 *Enterovirus* D68 strains available in GenBank as of October 2016. Phylogenetic tree was constructed by the unweighted pair-group method with arithmetic averages (UPGMA) clustering method using the BioNumerics software (version 7.6, Applied Maths, Belgium). Enterovirus strains EV-D70 and EV-D90 were used as outgroup. The numbers at each branch node are the % of nucleotide sequence identify. Country abbreviations: CAN: Canada; CHN: China; FRA: France; HTI: Haiti; JPN: Japan; MEX: Mexico; NLD: The Netherlands; NZL: New Zealand; THA: Thailand; TWN: Taiwan; USA: United States of America.

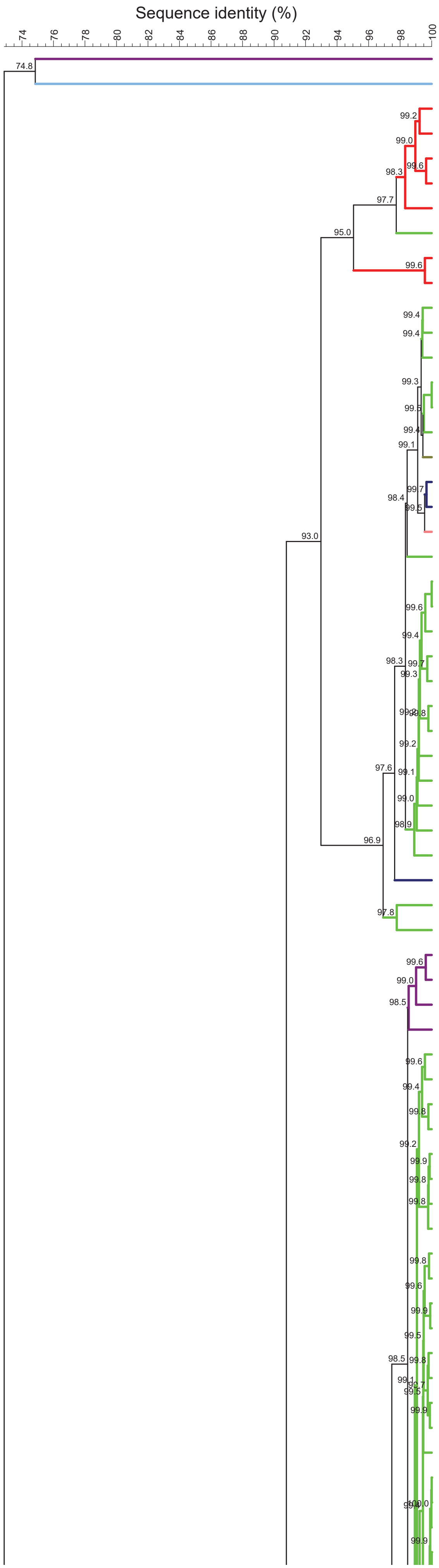

Accession#	Strain	Year	Country	Subtype	Length (bp)
DQ201177	ATCC VR-836		JPN	EV-D70	7335
DQ916376	E210		Egypt	EV-D94	7299
KT280501	2013-1017-26	2013	CHN	Clade D	7348
KT280502	2013-0825-6	2013	CHN	Clade D	7348
KT285319	2012-12225	2012	CHN	Clade D	7346
KT306743	2011-21286	2011	CHN	Clade D	7345
KT803588	CHN/CQ5508/2013	2013	CHN	Clade D	7039
KM851231	US/KY/14-18953	2014	USA	Clade D	7348
KF726085	BCH895A	2008	CHN	Clade D	7348
KU242683	BJ24	2008	CHN	Clade D	7326
KX255409	USA/U2752/2009	2009	USA	Clade A	7291
KX261804	USA/MO67/2009	2009	USA	Clade A	7293
KX261824	USA/MO94/2009	2009	USA	Clade A	7293
KX255360	USA/U2705/2009	2009	USA	Clade A	7291
KX255374	USA/U2704/2009	2009	USA	Clade A	7291
KX255355	USA/U2737/2009	2009	USA	Clade A	7291
JX070222	NZ-2010-541	2010	NZL	Clade A	7320
KT231905	4311000670_tMK3RD1	2010	NLD	Clade A	7100
KT231907	4311000742_tMK3RD1	2010	NLD	Clade A	7101
KM361525	CU70	2011	THA	Clade A	7018
JX101846	NYC403	2009	USA	Clade A	7341
KX255359	USA/O979a/2012	2012	USA	Clade A	7291
KX255396	USA/O980a/2012	2012	USA	Clade A	7291
KX255392	USA/C7396/2012	2012	USA	Clade A	7291
KX255380	USA/O527a/2012	2012	USA	Clade A	7291
KX255404	USA/O685a/2012	2012	USA	Clade A	7291
KX255358	USA/O1312a/2012	2012	USA	Clade A	7291
KX255369	USA/O541a/2012	2012	USA	Clade A	7291
KX255386	USA/O593a/2012	2012	USA	Clade A	7290
KX255381	USA/O433a/2012	2012	USA	Clade A	7291
KX255361	USA/O1261a/2012	2012	USA	Clade A	7291
KT347280	USA/N0051U5/2012	2012	USA	Clade A	7293
KX255376	USA/C7245/2011	2011	USA	Clade A	7291
KT231899	4310901348_RD2	2009	NLD	Clade A	6978
KM892500	CA/RESP/10-786	2010	USA	Clade A	7338
KX255363	USA/C6944/2006	2006	USA	Clade A	7291
AB601883	JPOC10-378	2010	JPN	Clade C	7328
AB601884	JPOC10-396	2010	JPN	Clade C	6764
AB601882	JPOC10-290	2010	JPN	Clade C	7329
AB601885	JPOC10-404	2010	JPN	Clade C	6764
KX255366	USA/U2215/2008	2008	USA	Clade C	7283
KX255371	USA/U2221/2008	2008	USA	Clade C	7284
KX261793	USA/MO68/2009	2009	USA	Clade C	7284
KX261814	USA/MO93/2009	2009	USA	Clade C	7284
KX261797	USA/MO74/2009	2009	USA	Clade C	7284
KX261805	USA/MO76/2009	2009	USA	Clade C	7284
KX261810	USA/MO81/2009	2009	USA	Clade C	7264
KX261811	USA/MO73/2009	2009	USA	Clade C	7284
KX261795	USA/MO70/2009	2009	USA	Clade C	7284
KX261803	USA/MO85/2009	2009	USA	Clade C	7284
KX261798	USA/MO79/2009	2009	USA	Clade C	7284
KX261802	USA/MO80/2009	2009	USA	Clade C	7284
KX255402	USA/C6832/2009	2009	USA	Clade C	7284
KX261820	USA/MO61/2009	2009	USA	Clade C	7284
KX261813	USA/MO92/2009	2009	USA	Clade C	7284
KX261815	USA/MO72/2009	2009	USA	Clade C	7284
KX261796	USA/MO89/2009	2009	USA	Clade C	7274
KX261807	USA/MO62/2009	2009	USA	Clade C	7284
KX261818	USA/MO86/2009	2009	USA	Clade C	7284
KX261794	USA/MO63/2009	2009	USA	Clade C	7284
KX261809	USA/MO83/2009	2009	USA	Clade C	7284

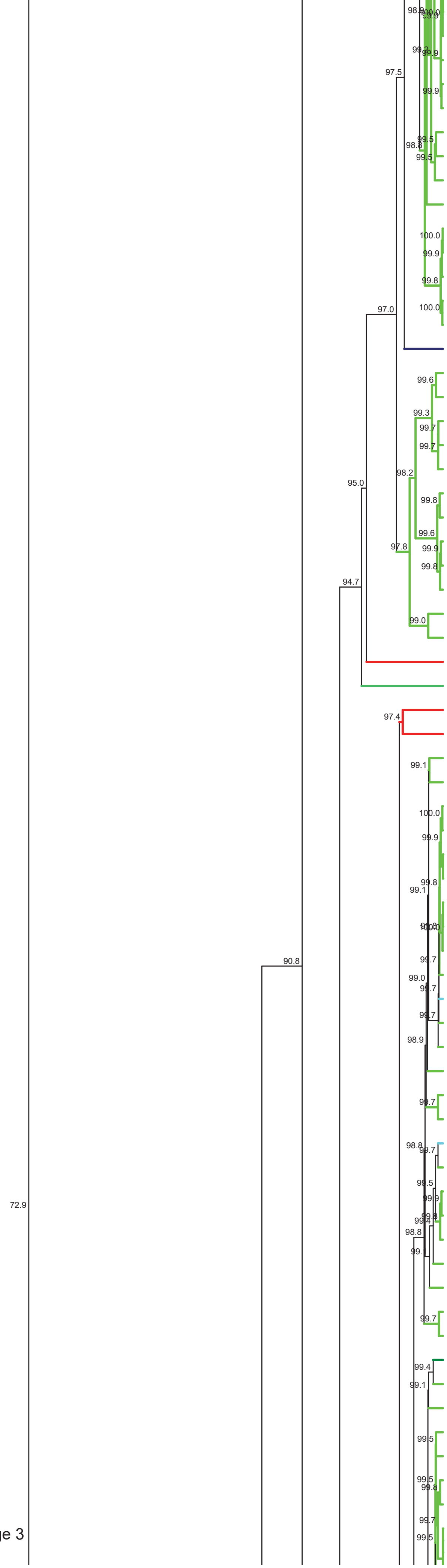

KX261819	USA/MO84/2009	2009	USA	Clade C	7284
KX261801	USA/MO82/2009	2009	USA	Clade C	7284
KX261812	USA/MO71/2009	2009	USA	Clade C	7284
KX261800	USA/MO90/2009	2009	USA	Clade C	7284
KX261808	USA/MO64/2009	2009	USA	Clade C	7284
KX261823	USA/MO87/2009	2009	USA	Clade C	7284
KX261826	USA/MO66/2009	2009	USA	Clade C	7284
KX261821	USA/MO91/2009	2009	USA	Clade C	7284
KX255408	USA/C6840/2009	2009	USA	Clade C	7284
KX261806	USA/MO75/2009	2009	USA	Clade C	7284
KX261822	USA/MO77/2009	2009	USA	Clade C	7284
KX261816	USA/MO88/2009	2009	USA	Clade C	7284
KX261799	USA/MO65/2009	2009	USA	Clade C	7284
KX261817	USA/MO69/2009	2009	USA	Clade C	7284
KT231903	4310902284_RD2	2009	NLD	Clade C	7093
KX255357	USA/C3578/2000	2000	USA	Clade C	7285
KX255391	USA/C6261/2000	2000	USA	Clade C	7285
KX255405	USA/C2590/2000	2000	USA	Clade C	7285
KX433166	USA/C6633/2000	2000	USA	Clade C	7285
KX255353	USA/C3963/2000	2000	USA	Clade C	7285
KX255372	USA/C2206/2003	2003	USA	Clade C	7285
KX255412	USA/O622a/2012	2012	USA	Clade C	7285
KX255388	USA/C2386/2003	2003	USA	Clade C	7285
KX255410	USA/C2204/2003	2003	USA	Clade C	7285
KX255400	USA/C3256/2003	2003	USA	Clade C	7285
KX255393	USA/C3486/2005	2005	USA	Clade C	7285
KX255397	USA/U807/2007	2007	USA	Clade C	7285
KT285484	BCH824A	2008	CHN	Clade C	7331
EF107098	FR/37-99	1999	FRA	Clade C	7333
KT280504	2013-0720-6	2013	CHN	Subclade B1	7330
KT803589	CHN/CQ2860/2012	2012	CHN	Subclade B1	7024
KP100794	US/CO/13-60	2013	USA	Subclade B1	7315
KX351812	USA/SSENT10/2014	2014	USA	Subclade B1	7285
KX255354	USA/C7731/2014	2014	USA	Subclade B1	7285
KX255385	USA/C7733/2014	2014	USA	Subclade B1	7285
KX255352	USA/C7720/2014	2014	USA	Subclade B1	7285
KX255415	USA/C7721/2014	2014	USA	Subclade B1	7285
KX255362	USA/C7715/2014	2014	USA	Subclade B1	7285
KX255390	USA/C7714/2014	2014	USA	Subclade B1	7285
KX255403	USA/C7716/2014	2014	USA	Subclade B1	7285
KX255378	USA/C7729/2014	2014	USA	Subclade B1	7285
KT835408	Ontario/C818712/2014	2014	CAN	Subclade B1	7292
KX255379	USA/C7793/2014	2014	USA	Subclade B1	7285
KX255411	USA/C7730/2014	2014	USA	Subclade B1	7285
KT347275	USA/MO35/2014	2014	USA	Subclade B1	7285
KP745756	NY160	2014	USA	Subclade B1	7294
KP745767	NY329	2014	USA	Subclade B1	7319
KP114663	Alberta4693_2014	2014	CAN	Subclade B1	6839
KT347258	USA/MO12/2014	2014	USA	Subclade B1	7285
KT347233	USA/MO19/2014	2014	USA	Subclade B1	7285
KT347278	USA/MO8/2014	2014	USA	Subclade B1	7285
KT347252	USA/MO51/2014	2014	USA	Subclade B1	7285
KT347254	USA/MO33/2014	2014	USA	Subclade B1	7285
KT455427	NYS14_31249	2014	USA	Subclade B1	6871
KT347247	USA/MO41/2014	2014	USA	Subclade B1	7285
KU844180	US/CA/14-4232	2014	USA	Subclade B1	7333
KT825142	MEX/DF/2014-InDRE2351	2014	MEX	Subclade B1	7319
KX351794	USA/SSENT29/2014	2014	USA	Subclade B1	7285
KX255375	USA/U5282/2014	2014	USA	Subclade B1	7285
KP745758	NY263	2014	USA	Subclade B1	7255
KX351797	USA/SSENT11/2014	2014	USA	Subclade B1	7285
KX351795	USA/SSENT27/2014	2014	USA	Subclade B1	7283
KX351798	USA/SSENT03/2014	2014	USA	Subclade B1	7285
KX255407	USA/C7743/2014	2014	USA	Subclade B1	7285
KX433161	USA/C7791/2014	2014	USA	Subclade B1	7285

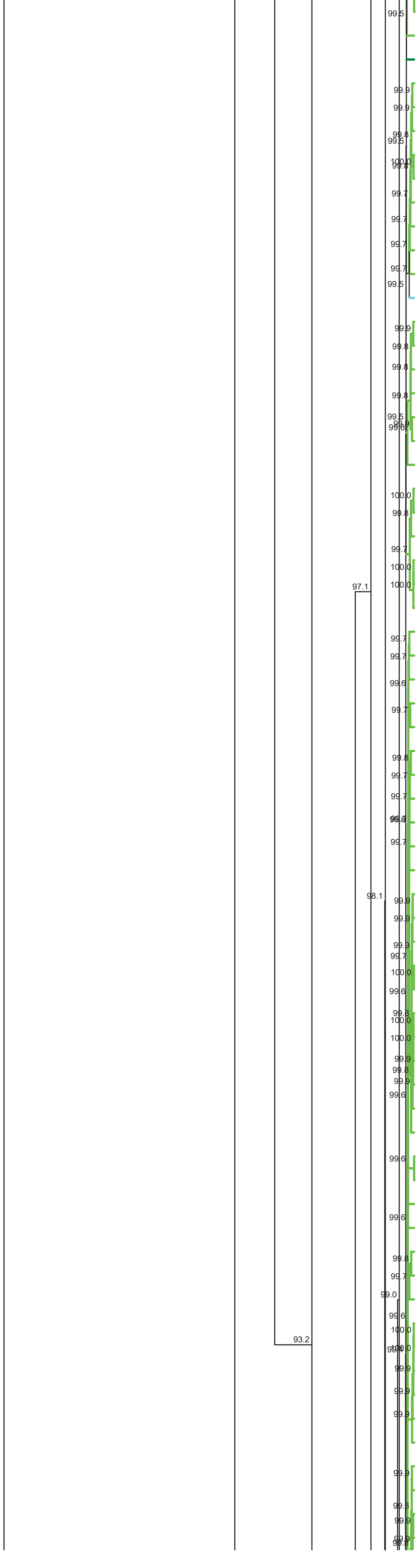

KX433167	USA/U797/2007	2007	USA	Subclade B1	7283
KP745759	NY275	2014	USA	Subclade B1	7312
KT803995	MEX/DGO/2014-InDRE2.	2014	MEX	Subclade B1	7319
KX351796	USA/SSENT21/2014	2014	USA	Subclade B1	7285
KX351801	USA/SSENT37/2014	2014	USA	Subclade B1	7285
KX351830	USA/SSENT24/2014	2014	USA	Subclade B1	7285
KP745751	NY120	2014	USA	Subclade B1	7319
KP745754	NY130	2014	USA	Subclade B1	7306
KX351820	USA/SSENT07/2014	2014	USA	Subclade B1	7285
KP745770	NY77	2014	USA	Subclade B1	7295
KP745757	NY210	2014	USA	Subclade B1	7319
KT347276	USA/MO21/2014	2014	USA	Subclade B1	7285
KT835407	Ontario/C818710/2014	2014	CAN	Subclade B1	7292
KT347253	USA/MO24/2014	2014	USA	Subclade B1	7285
KT347263	USA/MO28/2014	2014	USA	Subclade B1	7285
KT347251	USA/MO47/2014	2014	USA	Subclade B1	7285
KM851225	US/MO/14-18947	2014	USA	Subclade B1	7331
KM881710	STL_2014_12	2014	USA	Subclade B1	7296
KT347249	USA/MO16/2014	2014	USA	Subclade B1	7285
KT347260	USA/MO11/2014	2014	USA	Subclade B1	7285
KT347236	USA/MO39/2014	2014	USA	Subclade B1	7285
KT347279	USA/MO38/2014	2014	USA	Subclade B1	7285
KT347224	USA/MO58/2014	2014	USA	Subclade B1	7285
KX351810	USA/SSENT19/2014	2014	USA	Subclade B1	7285
KX351828	USA/SSENT04/2014	2014	USA	Subclade B1	7285
KX351829	USA/SSENT14/2014	2014	USA	Subclade B1	7283
KM851227	US/MO/14-18949	2014	USA	Subclade B1	7311
KT347238	USA/MO32/2014	2014	USA	Subclade B1	7286
KT347273	USA/MO42/2014	2014	USA	Subclade B1	7285
KT455425	NYS14_30905	2014	USA	Subclade B1	6868
KX351800	USA/SSENT31/2014	2014	USA	Subclade B1	7285
KM851228	US/MO/14-18950	2014	USA	Subclade B1	7292
KT347227	USA/MO26/2014	2014	USA	Subclade B1	7285
KT347248	USA/MO13/2014	2014	USA	Subclade B1	7285
KT347257	USA/MO10/2014	2014	USA	Subclade B1	7285
KX351813	USA/SSENT17/2014	2014	USA	Subclade B1	7285
KX351808	USA/SSENT25/2014	2014	USA	Subclade B1	7285
KX351805	USA/SSENT18/2014	2014	USA	Subclade B1	7283
KX351824	USA/SSENT05/2014	2014	USA	Subclade B1	7283
KX351819	USA/SSENT12/2014	2014	USA	Subclade B1	7283
KX351803	USA/SSENT22/2014	2014	USA	Subclade B1	7275
KX351814	USA/SSENT02/2014	2014	USA	Subclade B1	7285
KX351811	USA/SSENT08/2014	2014	USA	Subclade B1	7273
KX351816	USA/SSENT09/2014	2014	USA	Subclade B1	7285
KX351825	USA/SSENT28/2014	2014	USA	Subclade B1	7283
KX351822	USA/SSENT16/2014	2014	USA	Subclade B1	7285
KX351821	USA/SSENT39/2014	2014	USA	Subclade B1	7284
KX351817	USA/SSENT35/2014	2014	USA	Subclade B1	7285
KP100795	US/CA/14-6103SIB	2014	USA	Subclade B1	7285
KP100796	US/CA/14-6100	2014	USA	Subclade B1	7149
KP100792	US/CA/14-6092	2014	USA	Subclade B1	7024
KT347241	USA/MO18/2014	2014	USA	Subclade B1	7285
KT455430	NYS14_32368	2014	USA	Subclade B1	6868
KX351806	USA/SSENT36/2014	2014	USA	Subclade B1	7285
KT455428	NYS14_31590	2014	USA	Subclade B1	6868
KT347226	USA/MO53/2014	2014	USA	Subclade B1	7285
KT347272	USA/MO44/2014	2014	USA	Subclade B1	7285
KT347242	USA/MO5/2014	2014	USA	Subclade B1	7285
KT347250	USA/MO37/2014	2014	USA	Subclade B1	7285
KT347255	USA/MO23/2014	2014	USA	Subclade B1	7285
KT347237	USA/MO30/2014	2014	USA	Subclade B1	7285
KT455424	NYS14_30609	2014	USA	Subclade B1	6868
KX351802	USA/SSENT06/2014	2014	USA	Subclade B1	7282
KP100793	US/CO/14-94	2014	USA	Subclade B1	7265
KT347266	USA/MO27/2014	2014	USA	Subclade B1	7285

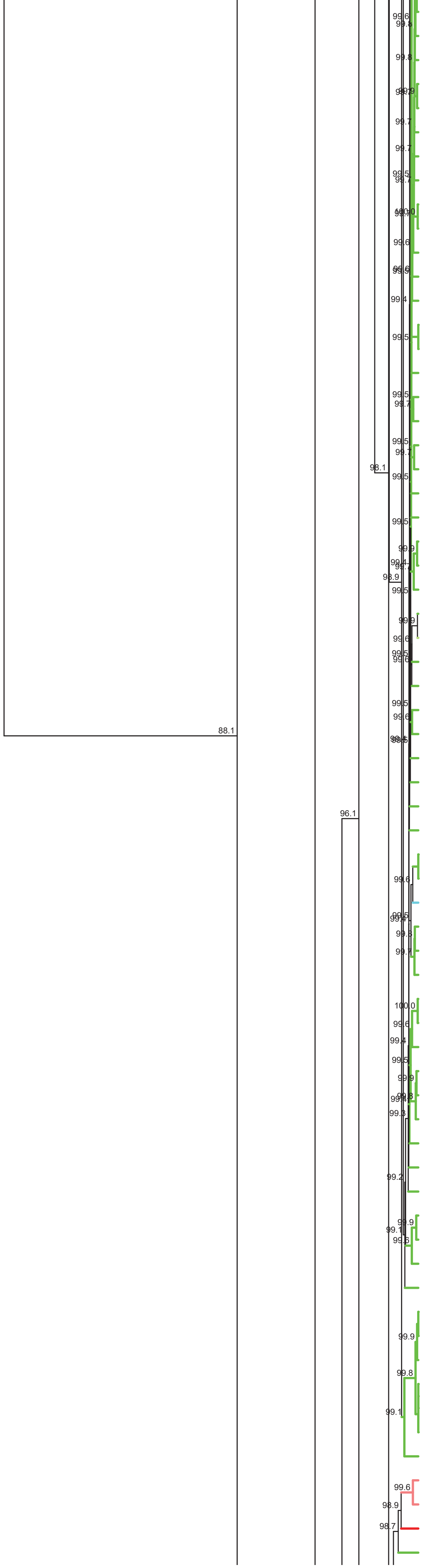

KT347235	USA/MO4/2014	2014	USA	Subclade B1	7285
KT347243	USA/MO14/2014	2014	USA	Subclade B1	7285
KT347229	USA/MO31/2014	2014	USA	Subclade B1	7285
KT347234	USA/MO1/2014	2014	USA	Subclade B1	7285
KT347277	USA/MO2/2014	2014	USA	Subclade B1	7285
KT347232	USA/MO46/2014	2014	USA	Subclade B1	7285
KT347256	USA/MO20/2014	2014	USA	Subclade B1	7285
KT347262	USA/MO57/2014	2014	USA	Subclade B1	7285
KT347230	USA/MO48/2014	2014	USA	Subclade B1	7285
KT347270	USA/MO17/2014	2014	USA	Subclade B1	7285
KT347246	USA/MO56/2014	2014	USA	Subclade B1	7285
KT455423	NYS14_30603	2014	USA	Subclade B1	6848
KX351815	USA/SSENT33/2014	2014	USA	Subclade B1	7285
KX255395	USA/C7789/2014	2014	USA	Subclade B1	7285
KX255413	USA/C7788/2014	2014	USA	Subclade B1	7285
KT347225	USA/MO25/2014	2014	USA	Subclade B1	7285
KT347244	USA/MO3/2014	2014	USA	Subclade B1	7285
KT455426	NYS14_30917	2014	USA	Subclade B1	6869
KT347239	USA/MO6/2014	2014	USA	Subclade B1	7285
KT347240	USA/MO49/2014	2014	USA	Subclade B1	7285
KT347265	USA/MO36/2014	2014	USA	Subclade B1	7286
KX255382	USA/C7787/2014	2014	USA	Subclade B1	7285
KP745764	NY316	2014	USA	Subclade B1	7320
KU509997	Gainesville/1/2015	2015	USA	Subclade B1	7320
KP745766	NY328	2014	USA	Subclade B1	7320
KP745755	NY153	2014	USA	Subclade B1	7319
KT266905	Haiti/1/2014	2014	HTI	Subclade B1	7319
KT347231	USA/MO40/2014	2014	USA	Subclade B1	6946
KP745760	NY278	2014	USA	Subclade B1	7319
KT347271	USA/MO7/2014	2014	USA	Subclade B1	7285
KX351827	USA/SSENT01/2014	2014	USA	Subclade B1	7285
KP745762	NY309	2014	USA	Subclade B1	7255
KP745765	NY326	2014	USA	Subclade B1	7294
KT347269	USA/MO45/2014	2014	USA	Subclade B1	7285
KP745761	NY305	2014	USA	Subclade B1	7312
KX255364	USA/C7722/2014	2014	USA	Subclade B1	7285
KX255373	USA/C7723/2014	2014	USA	Subclade B1	7285
KP114662	Alberta17390_2014	2014	CAN	Subclade B1	6933
KT347223	USA/MO54/2014	2014	USA	Subclade B1	7285
KT347259	USA/MO9/2014	2014	USA	Subclade B1	7285
KT347274	USA/MO43/2014	2014	USA	Subclade B1	7285
KT347228	USA/MO15/2014	2014	USA	Subclade B1	7285
KT347261	USA/MO60/2014	2014	USA	Subclade B1	7285
KX351804	USA/SSENT38/2014	2014	USA	Subclade B1	7283
KM851226	US/MO/14-18948	2014	USA	Subclade B1	7215
KT347245	USA/MO59/2014	2014	USA	Subclade B1	7285
KT347267	USA/MO34/2014	2014	USA	Subclade B1	7285
KT347264	USA/MO22/2014	2014	USA	Subclade B1	7285
KP745753	NY126	2014	USA	Subclade B1	7270
KX255389	USA/C7745/2014	2014	USA	Subclade B1	7283
KP745752	NY124	2014	USA	Subclade B1	7275
KP745763	NY314	2014	USA	Subclade B1	7295
KX351823	USA/SSENT23/2014	2014	USA	Subclade B1	7283
KX255367	USA/C7732/2014	2014	USA	Subclade B1	7285
KX255384	USA/O4413/2013	2013	USA	Subclade B1	7285
KX255406	USA/O4415/2013	2013	USA	Subclade B1	7285
KX255368	USA/O4417/2013	2013	USA	Subclade B1	7285
KX255387	USA/O4445/2013	2013	USA	Subclade B1	7285
KX255398	USA/O4446/2013	2013	USA	Subclade B1	7285
KX433165	USA/O4444/2013	2013	USA	Subclade B1	7283
KX255383	USA/O810a/2012	2012	USA	Subclade B1	7285
KM361523	CU134	2011	THA	Subclade B1	7010
KM361524	CU171	2011	THA	Subclade B1	7010
KT280503	2011-21186	2011	CHN	Subclade B1	7330
KM892501	CA/AFP/11-1767	2011	USA	Subclade B1	7341

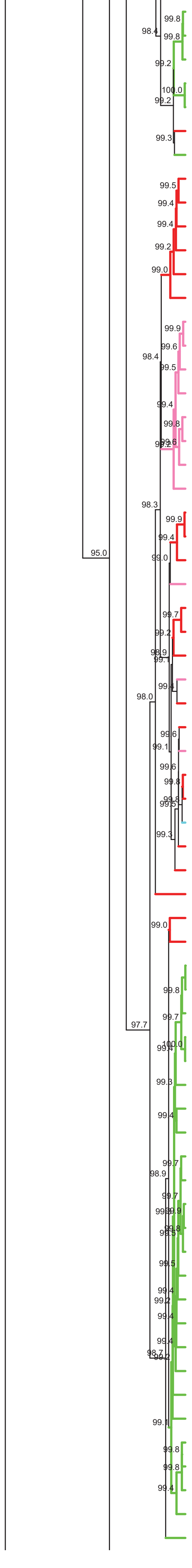

KX255356	USA/U4489/2012	2012	USA	Subclade B1	7285
KX255399	USA/U4486/2012	2012	USA	Subclade B1	7285
KX255370	USA/U4470/2012	2012	USA	Subclade B1	7285
KX255377	USA/O774a/2012	2012	USA	Subclade B1	7285
KX255414	USA/O775a/2012	2012	USA	Subclade B1	7285
KT285320	2011-21282	2011	CHN	Subclade B1	7330
KX255365	USA/O466a/2012	2012	USA	Subclade B1	7285
KT803593	CHN/CQ7208/2014	2014	CHN	Subclade B3	7024
KT803595	CHN/CQ7280/2014	2014	CHN	Subclade B3	7024
KT803597	CHN/CQ7233/2014	2014	CHN	Subclade B3	7024
KT803590	CHN/CQ5571/2013	2013	CHN	Subclade B3	7024
KT280499	2014-R970	2014	CHN	Subclade B3	7330
KT803591	CHN/CQ7170/2014	2014	CHN	Subclade B3	7024
KT711078	TW-00880-2014	2014	TWN	Subclade B3	7333
KT711083	TW-00785-2014	2014	TWN	Subclade B3	7330
KT711082	TW-00898-2014	2014	TWN	Subclade B3	7329
KT711079	TW-00893-2014	2014	TWN	Subclade B3	7330
KT711081	TW-00932-2014	2014	TWN	Subclade B3	7330
KT711085	TW-00928-2014	2014	TWN	Subclade B3	7330
KT711084	TW-00821-2014	2014	TWN	Subclade B3	7330
KT711080	TW-00909-2014	2014	TWN	Subclade B3	7330
KP240936	Beijing-R0132	2014	CHN	Subclade B3	7330
KT285485	BCH4235A	2016	CHN	Subclade B3	7330
KT280496	2014-R1357	2014	CHN	Subclade B3	7330
KT711086	TW-02512-2014	2014	TWN	Subclade B3	7330
KT280500	2014-R0672	2014	CHN	Subclade B3	7330
KT803592	CHN/CQ7188/2014	2014	CHN	Subclade B3	7024
KT803598	CHN/CQ7174/2014	2014	CHN	Subclade B3	7024
KT711087	TW-02809-2014	2014	TWN	Subclade B3	7330
KT803600	CHN/CQ7360/2014	2014	CHN	Subclade B3	7024
KT280498	2014-R1011	2014	CHN	Subclade B3	7330
KT711088	TW-02795-2014	2014	TWN	Subclade B3	7330
KT803594	CHN/CQ7214/2014	2014	CHN	Subclade B3	7024
KT803596	CHN/CQ7226/2014	2014	CHN	Subclade B3	7024
KP114664	Alberta2985_2014	2014	CAN	Subclade B3	6902
KT803599	CHN/CQ7225/2014	2014	CHN	Subclade B3	7024
KT280497	2014-R1153	2014	CHN	Subclade B3	7330
KU982561	SZ04/CHN/2015	2015	CHN	Subclade B3	7291
KU982558	SZ01/CHN/2015	2015	CHN	Subclade B3	7291
KU982559	SZ02/CHN/2015	2015	CHN	Subclade B3	7291
KX957757	NY30_16	2016	USA	Subclade B3	7331
KX957760	NY44_16	2016	USA	Subclade B3	7310
KY385889	NY212_16	2016	USA	Subclade B3	7333
KX957756	NY29_16	2016	USA	Subclade B3	7331
KX957759	NY43_16	2016	USA	Subclade B3	7331
KY385880	NY126_16	2016	USA	Subclade B3	7290
KX957755	NY22_16	2016	USA	Subclade B3	7304
KY385884	NY151_16	2016	USA	Subclade B3	7325
KX675261	USA/FL/2016-19504	2016	USA	Subclade B3	7331
KX675263	USA/TX/2016-19506	2016	USA	Subclade B3	7310
KY385886	NY172_16	2016	USA	Subclade B3	7300
KY385890	NY230_16	2016	USA	Subclade B3	7332
KY385882	NY141_16	2016	USA	Subclade B3	7334
KX957758	NY39_16	2016	USA	Subclade B3	7332
KY385887	NY185_16	2016	USA	Subclade B3	7332
KY385881	NY135_16	2016	USA	Subclade B3	7332
KY385888	NY188_16	2016	USA	Subclade B3	7334
KX957762	NY75_16	2016	USA	Subclade B3	7306
KY385885	NY166_16	2016	USA	Subclade B3	7326
KX957754	NY10_16	2016	USA	Subclade B3	7321
KX957761	NY59_16	2016	USA	Subclade B3	7321
KY385892	NY241_16	2016	USA	Subclade B3	7326
KY385883	NY149_16	2016	USA	Subclade B3	7320
KY385891	NY234_16	2016	USA	Subclade B3	7315
KX675262	USA/NY/2016-19505	2016	USA	Subclade B3	7319

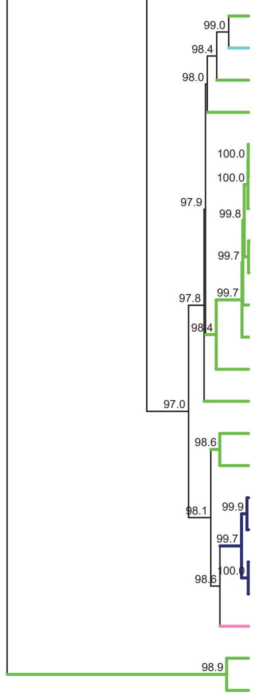

KM851230	US/IL/14-18952	2014	USA	Subclade B2	6866
KP114665	Alberta17789_2014	2014	CAN	Subclade B2	6906
KM851229	US/KY/14-18951	2014	USA	Subclade B2	6869
KX255394	USA/O418a/2012	2012	USA	Subclade B2	7282
KP745768	NY73	2014	USA	Subclade B2	7289
KP745769	NY74	2014	USA	Subclade B2	7219
KT455422	NYS14_28173	2014	USA	Subclade B2	6865
KX261825	USA/MO78/2009	2009	USA	Subclade B2	7282
KX351818	USA/SSENT20/2014	2014	USA	Subclade B2	7282
KU844181	US/CA/14-4231	2014	USA	Subclade B2	7327
KT455429	NYS14_32132	2014	USA	Subclade B2	6865
KX351799	USA/SSENT30/2014	2014	USA	Subclade B2	7282
KX351807	USA/SSENT32/2014	2014	USA	Subclade B2	7282
KX255401	USA/O270a/2012	2012	USA	Subclade B2	7285
KX433164	USA/C7413/2012	2012	USA	Subclade B2	7285
KT231901	4310902042_RD2	2009	NLD	Subclade B2	7058
KT231902	4310902042_RD3	2009	NLD	Subclade B2	7292
KT231897	4310900947_RD2	2009	NLD	Subclade B2	7006
KT231898	4310900947_RD3	2009	NLD	Subclade B2	7292
KT318494	1-I8/TW/2008	2008	TWN	Subclade B2	7330
AY426531	Fermon	1962	USA	Prototype	7367
KU844178	US/CA/Rhyne	1962	USA	Prototype	7317
